# Circulating tumor DNA in patients with cancer: insights from clinical laboratory

**DOI:** 10.1515/almed-2025-0010

**Published:** 2025-06-16

**Authors:** Francisco J. Illana, Esther Fernández-Galán, José Luis Muñoz-Bravo, Laura Valiña Amado, Carme García Martín, Carolina González-Fernández, Sílvia Miró-Cañís, Jaume Trapé, Antonio Martínez-Peinado, Xavier Filella, Alvaro González, Antonio Barco Sánchez, Angel Díaz-Lagares, On behalf of the Commission on Biological Markers of Cancer of the Spanish Society of Laboratory Medicine

**Affiliations:** Department of Biochemistry, Hospital de la Santa Creu i Sant Pau, IIB Sant Pau, Barcelona, Spain; Service of Biochemistry and Molecular Genetics, CDB, Hospital Clínic de Barcelona, IDIBAPS, University of Barcelona, Barcelona, Spain; Clinical Analysis Service, General University Hospital of Elche, Elche, Spain; Foundation for the Promotion of Health and Biomedical Research in the Valencia Region (FISABIO), Elche, Spain; Department of Laboratory Medicine, Hospital Universitari Son Espases, Palma, Spain; Group of Advanced Therapies and Biomarkers in Clinical Oncology, Institut d’Investigació Sanitària de les Illes Balears (IdISBa), Palma, Spain; Clinical Analysis Laboratory, Biochemistry and Immunoassay Department, Germans Trias i Pujol University Hospital, Badalona, Spain; Department of Laboratory Medicine, Althaia Xarxa Assistencial Universitària de Manresa, Manresa, Catalonia, Spain; Clinical Analysis Laboratory, CLILAB Diagnòstics, Vilafranca del Penedès, Spain; Clinical Analysis Management Unit, Section of Molecular Genetics, Reina Sofía University Hospital, Córdoba, Spain; Department of Biochemistry, Clínica Universidad de Navarra, Pamplona, Spain; Department of Clinical Biochemistry, Virgen Macarena University Hospital, Seville, Spain; Department of Clinical Analysis, University Hospital Complex of Santiago de Compostela (CHUS), Santiago de Compostela, Spain; Epigenomics Unit, Cancer Epigenomics, Translational Medical Oncology Group (ONCOMET), Health Research Institute of Santiago de Compostela (IDIS), Santiago de Compostela, Spain; Centro de Investigación Biomédica en Red Cáncer (CIBERONC), ISCIII, Madrid, Spain

**Keywords:** biomarkers, cancer, ctDNA, liquid biopsy, somatic gene variants

## Abstract

Blood-based circulating tumor DNA (ctDNA) analysis has emerged as a highly relevant non-invasive method for molecular profiling of solid tumors, offering valuable information about the genetic landscape of cancer. Somatic mutation analysis of ctDNA is now used clinically to guide targeted therapies for advanced cancers. Recent advancements have also revealed its potential in early detection, prognosis, minimal residual disease assessment, and prediction/monitoring of therapeutic response. In recent years, significant progress has been made with the development of various PCR and NGS-based methods designed for assessing gene variants in ctDNA of patients with cancer. However, despite the transformative possibilities that ctDNA analysis presents, challenges persist. Standardization of preanalytical and analytical protocols, assay sensitivity, and the interpretation of results remain critical hurdles that need to be addressed for the widespread clinical implementation of ctDNA testing. In addition to somatic mutations, emerging studies on DNA methylation (epigenomics) and fragment size patterns (fragmentomics) in several types of biological fluids are yielding promising results as non-invasive biomarkers for effective cancer management. This review addresses the clinical applications of somatic gene variants in ctDNA, emphasizes their potential as cancer biomarkers, and highlights essential factors for successful implementation in clinical laboratories and cancer management.

## Introduction

**Figure 1:**
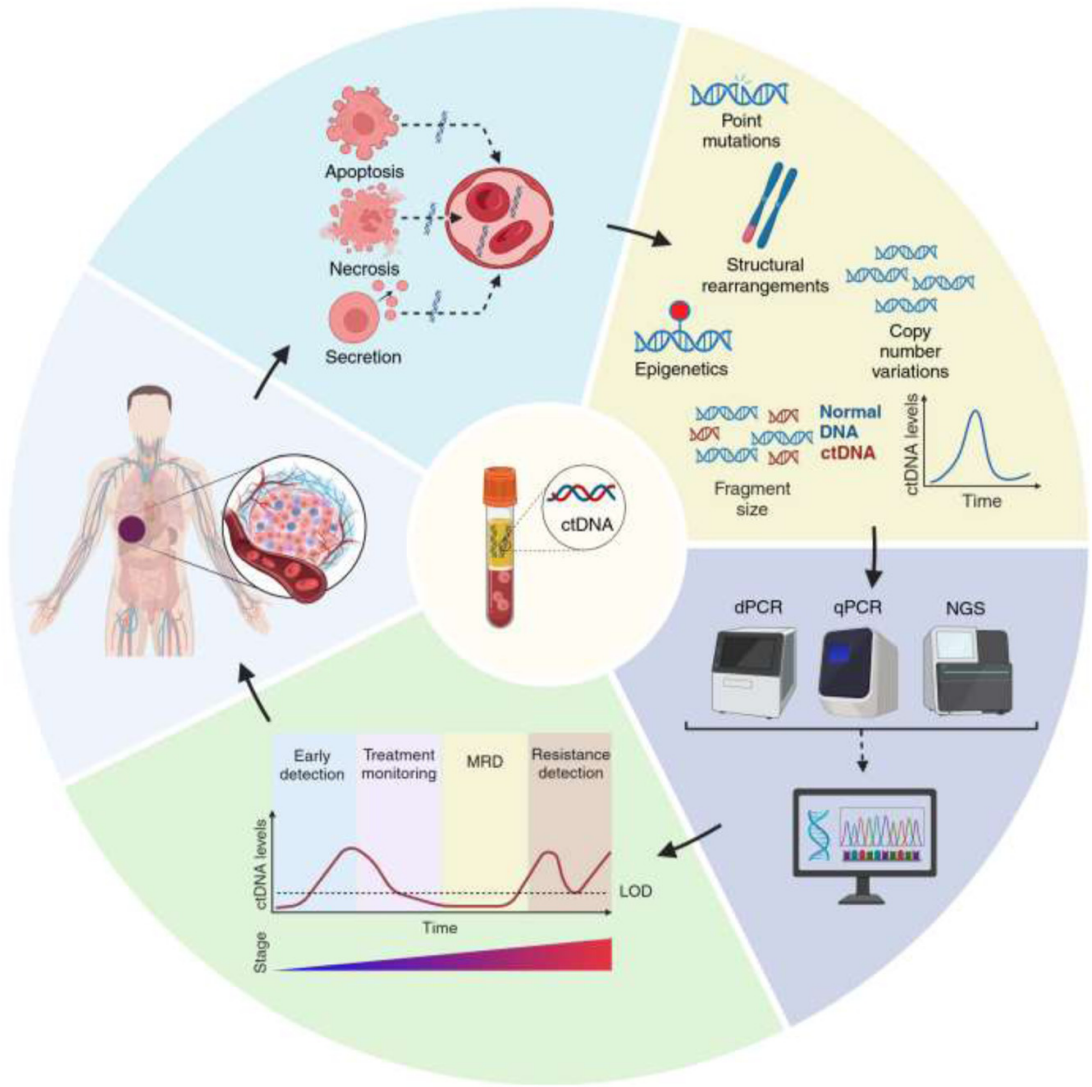
Schematic representation of ctDNA analysis in cancer patients. Solid tumors release ctDNA into circulation (apoptosis, necrosis, secretion), which carries specific genetic and epigenetic alterations representative of tumor molecular landscape. DNA fragments in circulation allow for potential evaluation of ctDNA features, including point mutations, structural rearrangements, copy number variations, epigenetics (DNA methylation), fragment size, and ctDNA levels. Current molecular assays for the study of ctDNA offers the possibility of analysing specific somatic genetic alterations using dPCR or qPCR, or molecular genotyping with NGS-based technologies. Potential clinical aplications in oncology encompass screening/early cancer detection, treatment monitoring, detection of MDR, and resistance detection. CtDNA, circulating tumor DNA; dPCR, digital PCR; qPCR, quantitative PCR; NGS, next-generation sequencing; LOD limit of detection, MRD, minimal residual disease. Figure created with biorender (https://BioRender.com/x96k221).

In recent years liquid biopsy has emerged as a non-invasive method for molecular tumor profiling through the analysis of circulating tumor components in several biological fluids, primarily in plasma (Figure 1) [1], [2]. This approach offers significant potential for cancer management, including early detection and screening, prognosis, minimal residual disease (MRD) detection, and monitoring of therapy response [1], [3]. Among the components analyzed, circulating tumor DNA (ctDNA) stands out for its ability to detect somatic mutations, providing valuable clinical insights [4]. Although somatic mutations are the most advanced molecular biomarkers for clinical implementation, other ctDNA features, such as DNA methylation [5] and fragment size patterns [6], are providing promising results for cancer management. Recent advancements in ctDNA assays have demonstrated their potential to guide targeted therapies for advanced cancers, with growing evidence supporting their integration into routine clinical practice. However, there are still relevant challenges in the field, such as standardization, preanalytical and analytical procedures, and result interpretation [4]. This review highlights the clinical applications of ctDNA, emphasizing its potential as a cancer biomarker. It also addresses preanalytical and analytical factors, detection assays, result interpretation and reporting aspects, as well as alternative fluids for ctDNA analysis. Altogether, we provide an overview of the clinical utility of ctDNA, and discuss challenges and future opportunities for its implementation in clinical practice.

## Circulating DNA

### Characteristics of blood-based ctDNA

Cell-free DNA (cfDNA) consists of nuclear and mitochondrial small fragments of double-strand DNA [7], which is mainly released into bloodstream by the haematological system in healthy individuals [8]. These DNA fragments range from approximately 40 to 200 base pairs (bp) in length, with a peak around 166 bp, corresponding with nucleosome-associated DNA fragments [7], [9]. In patients with cancer, cfDNA also contains a small subset of shorter fragments (∼145 bp) released into bloodstream by tumor cells known as ctDNA [10], which contributes to the elevated cfDNA levels observed in patients with cancer [11]. ctDNA is usually more fragmented than cfDNA [12] and it is characterised by presenting specific genetic and epigenetic features that provide information about the tumor of origin [10]. The release of ctDNA into circulation can be produced through various mechanisms (Figure 1), including passive and active processes [12], [13]. Passive release of ctDNA primarily occurs through apoptosis and necrosis [13]. Apoptosis is thought to be the major mechanism of shedding of ctDNA in most cancers, leading to a ladder-like pattern of DNA fragments [14] with a periodicity of 10 bp [11]. Although necrosis has a variable contribution to ctDNA shedding, this mechanism usually releases larger fragments of ctDNA, mainly >200 bp and even longer than 10.000 bp, along with an oligonucleosomal ladder-like pattern [13], [15], [16]. In addition, ctDNA can also be actively released via extracellular vesicles (e.g. exosomes, microvesicles or apoptotic bodies) [17], representing a promising area of active research [18]. However, its exact contribution to ctDNA is still under debate [19], [20]. On the other hand, it is important to note that ctDNA has a short half-life in circulation (less than 2 h) [21], which is influenced by enzymatic cleavage in the bloodstream, its clearance by the liver, and to a lesser extent, by the kidney [22]. Classically, the gold standard for molecular profiling of solid tumors has been the analysis of direct tissue biopsy. Nevertheless, in the last decade, blood-based analysis of ctDNA has emerged as a useful alternative [12], with increasing clinical evidence supporting its utility for the non-invasive molecular profiling of solid tumors [23], [24], [25], [26], [27]. ctDNA offers an opportunity for non-invasive analysis of tumors, providing the possibility of serial sampling to follow tumor evolution, with faster turnaround times, lower costs, and a more simplified process compared to tissue biopsy. Moreover, ctDNA allows the analysis of clonal evolution or mutational load of the tumor [28], and can capture heterogeneity better than tissue biopsy, which is inherently localized, and in some cancers difficult to obtain [12], [29]. In fact, ctDNA is able to capture tumor heterogeneity with up to 80–90 % sensitivity [30], depending on anatomical localization, and ctDNA abundance [31].

The analysis of ctDNA features can be useful for detecting a wide range of tumor characteristics (Figure 1) [4], [12]. Most publications have focused on the analysis of gene variants, such as single nucleotide variants (SNVs), insertions and deletions (indels), copy number variations (CNVs), and fusions. However, additional features of ctDNA have recently gained significant attention [12]. For example, ctDNA levels correlate with tumor size and can be used to assess disease progression [7], [28] with lower levels associated with better outcomes [21]. In addition, the fragment size pattern analysis of cfDNA and ctDNA in patients with cancer can potentially provide valuable information about the tissue of origin [32], and it can be applied to improve ctDNA detection [33]. Importantly, the methylation profile of ctDNA can also offer valuable information about the tumor [5], [34], such as tissue of origin [12], [35].

### ctDNA in non-blood body fluids

In addition to blood, ctDNA can be analyzed in alternative biological fluids, which may offer increased sensitivity in certain situations [36], [37], with distinct benefits and limitations [7], [37]. Nonetheless, its applicability in routine clinical analysis is hampered by its lack of standardization.

Urine is a promising non-invasive source of ctDNA for the detection of genitourinary tumors [38], [39], [40], [41], since it can improve the detection and management of these tumor types [42], [43], [44]. Another non-invasive source of ctDNA is saliva, allowing serial sampling for the analysis of head and neck tumors [45], [46], [47], [48]. Some studies suggest salivary ctDNA as the preferred sample for oral tumors, which may also be used in combination with blood-based ctDNA for other head and neck tumors [37], [49]. On the other hand, cerebrospinal fluid (CSF), is especially valuable for diagnosis and tracking of central nervous system primary cancers and intracranial metastasis [50], [51], [52], [53], [54]. Since the blood-brain barrier severely limits the passage of ctDNA between the bloodstream and the central nervous system [55], cerebrospinal fluid (CSF) represents a specific potential source of intracranial ctDNA [37]. This approach eliminates the need for brain tumor biopsy and allows serial sampling [56]. In addition to these fluids, the analysis of ctDNA in effusions can also provide some advantages, since these fluids present a better ratio of ctDNA-cfDNA than blood for proximal tumors [57]. For example, pleural ctDNA can offer fast detection of actionable mutations in lung cancer with more sensitivity than blood ctDNA [37]. Whereas peritoneal ctDNA has proven to be useful in the detection of peritoneal metastatic disease [37], [58]. Other types of fluid sources, such as bronchial lavages, offer the possibility of ctDNA analysis, providing valuable opportunities for lung cancer diagnostics [59]. This type of fluid contains higher ctDNA levels than blood, making it valuable for molecular profiling of lung tumors [59], [60], [61]. Furthermore, other potential sources of ctDNA with future clinical applicability are under investigation, including bile for pancreatic cancer [62], and breast milk from pregnant and postpartum women for breast cancer [63], among others.

## Preanalytical considerations of ctDNA analysis

Preanalytical considerations (Table 1) are critical for the reliable analysis of ctDNA, as improper handling or processing can lead to its contamination, degradation, or low yield [4], [64].

**Table 1:** Preanalytical considerations for ctDNA analysis.

Step	Considerations
Sample type	Plasma rather than serum is recommended.
Blood collection	EDTA tubes require processing within 2–4 h. Cell preservation tubes maintain sample integrity for several days at room temperature.
Blood transport	Agitation and temperature fluctuation should be avoided. EDTA tubes should arrive the laboratory before 2–4 h. Cell preservation tubes can be transported at room temperature for up to several days without significant degradation. For longer transport times, plasma should be separated and frozen.
Plasma separation and QC	Typically involves two centrifugation steps. Plasma should be obtained without disturbing the buffy coat or red blood cells. Hemolysis, lipemia, and icterus shoud be avoided.
Plasma storage conditions	For long-term storage (months): −80 °C. For short-term storage (up to 30 days): −20 °C. Avoid repeated freeze-thaw cycles that can lead to ctDNA fragmentation and a loss of analytical sensitivity.
Extraction methods	Manual or automated extraction methods can be used. Yield and purity is relevant for choosing the methodology.
QC and storage of ctDNA	Fluorometric or quantitative PCR are usually used to determine ctDNA concentration. CtDNA is usually store at −80 °C if not used immediately, and repeated freeze-thaw cycles should be avoided.

QC, quality control.

### Specimen types

For ctDNA analysis, plasma is preferred over serum. The coagulation process in serum can release genomic DNA (gDNA) from leukocytes, increasing contamination, and complicating the detection of low-frequency mutations. Plasma minimizes gDNA contamination and provides more reliable results for detecting low-abundance alterations [65].

### Blood collection and transport

It is essential to consider the timing of blood collection depending on the specific application of ctDNA analysis. Collecting the sample before treatment helps establish a baseline for ctDNA and initial tumor burden, during treatment contributes to monitor therapeutic efficacy and detect potential resistance, and after treatment enables the early identification of recurrences and progression [5], [21], [66]. For blood collection, tubes with or without preservatives can be used [67]. Among the tubes without preservatives, those containing potassium ethylenediaminetetraacetate (K2EDTA) as an anticoagulant are the preferred choice for the analysis of ctDNA. Of note, blood with K2EDTA tubes require to be processed within 2–4 h after extraction to prevent cell lysis and gDNA contamination. On the other hand, tubes with preservatives (e.g. Streck) are specifically designed to stabilize nucleated blood cells, allowing for the preservation of ctDNA in collected blood samples for up to several days at room temperature [1], [68], [69]. Blood tubes must be transported to the laboratory without agitation and protected from temperature fluctuations to prevent hemolysis and cellular damage [70]. When working with external laboratories, it’s important to consider the use of cell preservation tubes and adhere to proper storage times and temperatures [64].

### Plasma separation and quality control

Plasma separation typically involves two centrifugation steps: ∼1,600 × g at 4 °C for 10 min, and a second centrifugation at ∼16,000 × g at 4 °C for 10 min to obtain cell-free plasma [5]. This process helps eliminate cellular debris and improves ctDNA purity [65]. Factors like hemolysis, lipemia, and icterus can affect ctDNA analysis. Therefore, visual inspection of plasma after separation is recommended. To minimize hemolysis, gentle venipuncture and immediate inversion of collection tubes are advised [4], [64]. It is recommended to reject samples with hemolysis [67], [70], since it can promote the release of gDNA, interfering with the extraction process and reducing the proportion of ctDNA [67], [71]. Regarding lipemia and icterus, additional studies are required to determine the effect of elevated bilirubin levels, or hyperlipidemia impact on ctDNA levels.

### Plasma storage conditions

Plasma can be stored at −20 °C for up to 30 days if analysis is to be performed soon [1], [65], but it should be kept at −80 °C for long-term storage. Proper aliquoting is essential to avoid repeated freeze-thaw cycles, which can fragment DNA and reduce ctDNA yield, impacting assay accuracy [72].

### Extraction methods of ctDNA

Extraction methods should be tailored to the characteristics of ctDNA, which is typically found in low concentrations and as small fragments. Several commercial kits are available for ctDNA extraction, ensuring good recovery and reproducibility [65]. Laboratories should choose the most appropriate method, considering both yield and purity for low-molecular-weight DNA. Manual or automated procedures may be used, depending on the platform’s performance and the specific needs of the laboratory [64].

### Quality assessment and storage of ctDNA

Assessing ctDNA quality is critical for downstream analyses. Fluorometric quantification is usually used to measure ctDNA concentration, while electrophoresis-based methods are useful to verify cfDNA fragment size and confirm the absence of gDNA contamination. When not used immediately, ctDNA should be stored at −80 °C in multiple aliquots to prevent degradation from repeated freeze-thaw cycles [72], [73].

## Analytical considerations of ctDNA analysis

When analyzing ctDNA, we must keep in mind several analytical factors (Table 2) that often interfere with the results of somatic variants, such as clonal hematopoiesis, and the presence of germline variants. Additionally, it is important to consider the use of both internal and external quality controls, and to conduct thorough analytical validation to optimize ctDNA detection in routine practice, ensuring that assays are reliable and clinically applicable.

**Table 2:** Analytical considerations for ctDNA assays.

Feature	Considerations
Clonal hematopoiesis (CHIP)	CHIP complicates ctDNA interpretation by potentially generating false positives. Sequencing of PBMCs avoids confounding results from CHIP.
Internal and external QC	Internal and external QC help assess the quality of ctDNA analysis.
Germline variants	Pathogenic germline variants in cancer predisposition genes can be detected through ctDNA testing.
Analytical validation	Analytical sensitivity (limit of detection) and analytical accuracy are key analytical performance parameters to evaluate for validation of ctDNA assays.
Analytical limitations	Limited sensitivity respect to tissue genotyping due to: i) low VAF and high fragmentation of ctDNA, ii) clonal heterogeneity, characterized by multiple tumor clones with different mutations at low VAF, iii) elevated levels of cfDNA that dilute ctDNA, and iii) limited shedding in early-stage cancers and low tumor burden.

QC, quality control; PBMCs, peripheral blood mononuclear cells.

### Clonal hematopoiesis

Clonal hematopoiesis of indeterminate potential (CHIP) is an age-related process in which somatic mutations in hematopoietic stem cells cause clonal expansion [4], [74] As cfDNA largely originates from hematopoietic cells, CHIP complicates ctDNA interpretation by potentially generating false positives, particularly in genes typically associated with solid tumors, including *KRAS*, *GNAS*, *NRAS*, and *PIK3CA* [75]. For accurate variant interpretation, it is important to properly differentiate between CHIP-related and tumor-derived mutations, which usually implies sequencing of both cfDNA and peripheral blood mononuclear cells (PBMCs) [76]. Approaches based on analyzing cfDNA fragment size also may improve the accuracy of variant interpretation [33], [77].

### Germline variants

Incidental detection of pathogenic germline variants (PGVs) should be considered when evaluating ctDNA results, particularly in NGS-based tests that include cancer predisposition genes (such as *BRCA1*, *BRCA2*, *PALB2*) [4], [78], [79]. In this regard, the presence of a variant allelic frequency (VAF) in circulation between 40 % and 60 % suggests germline origin, while somatic variants typically have lower VAFs [80]. However, we also have to take in mind that ctDNA may show increased VAFs due to the presence of high tumor burden or loss-of-heterozygosity [78], [81]. Only variants classified as Pathogenic and Likely Pathogenic according to American College of Medical Genetics and Genomics guidelines [82], ClinVar, and other sources, should be considered as potential PGV [79]. According to European Society for Medical Oncology (ESMO) recommendations, when a potential germline variant is suspected, reflex germline testing with a validated assay should be carried out to confirm their germline or somatic origin [4]. Consequently, it is essential to alert clinicians to the potential detection of germline mutations or those related to CHIP, particularly in ctDNA assays that target frequently mutated genes.

### External and internal QC

The implementation of internal and external QCs is crucial to ensure accuracy and reproducibility in detecting gene variants in ctDNA from patients with cancer. Internal controls, such as the Structural Multiplex cfDNA Reference Standard (HD786) from Horizon Discovery, are commercially available and help assess the quality of ctDNA analysis [83]. Additionally, external quality assessments (EQA), like those offered for example by the European Molecular Genetics Quality Network (EMQN), provide quality evaluation programs for ctDNA variant detection (e.g., “LUNG CANCER (NSCLC) [Plasma],” “cfDNA Multiple Biomarkers”). The use of these controls can help identify technical errors and ensure reliable clinical results, essential for biomarker-driven therapeutic decisions. Continuous validation through internal standards and comparisons with external proficiency schemes ensures the robustness of ctDNA analysis [83], [84], [85].

### Analytical validation

Analytical validation must be established to optimize ctDNA detection in routine clinical practice, and it should be tailored to the specific patient population and the medical indication for the test. Recommendations and protocols for ctDNA assay validation include evaluating analytical performance parameters, such as analytical sensitivity, accuracy, repeatability, precision, and reproducibility [86], [87]. Laboratories should define and evaluate the limit of detection for at least each variant class to ensure reliable results at low frequencies [86]. Analytical accuracy can be assessed by method comparison (comparing results to an orthogonal method) or with known reference standards [87]. Orthogonal assay confirmations for analytical validation may include quantitative PCR, digital PCR (dPCR), droplet digital PCR (ddPCR), NGS, or any method with sensitivity equal to or greater than that of the assay being validated [86].

### Analytical limitations

The primary limitation of ctDNA analysis is its lower sensitivity compared to tissue genotyping, leading to a higher rate of false negatives. This reduced sensitivity stems from several factors, including the extremely low concentration and high fragmentation of cfDNA in plasma, as well as the low proportion of ctDNA within the total cfDNA pool, typically ranging from 0.01 to 0.1 %. Additionally, clonal heterogeneity and elevated concentration of normal cfDNA, often arising from non-malignant conditions or postoperative inflammation, further dilute ctDNA, making the detection of low-frequency variants more challenging [88], [89]. Detection reliability is especially compromised for mutations with low VAFs. Other contributing factors include early tumor stage, low tumor burden, and non-shedding tumors, all of which can reduce detection rates. The quantity of cfDNA input is also a critical variable, since higher input is associated with enhanced fragment depth, sensitivity, and reproducibility [90]. On the other hand, the detection of false positives in ctDNA analysis is relatively rare. Although false positives tend to occur in low-frequency variants, they can be minimized by employing unique molecular identifiers (UMIs) and setting a minimum VAF threshold, typically above 0.05 %, that reduces sequencing error impact [90].

## Methods for the analysis of gene variants in ctDNA

In recent years, both the scientific community and diagnostic companies have developed multiple methodologies to study ctDNA in solid tumors (Figure 1). However, the complexity and limitations of these molecular analyses have confined their use primarily to clinical research, with only a few approved for *in vitro* diagnostic (IVD). Although several regulatory-approved tests are available for outsourcing to private foreign laboratories (e.g., Guardant360 CDx, FoundationOne Liquid CDx) for various clinical applications [91], we will focus on tests and technologies that can be integrated into clinical laboratories for routine use. Table 3 summarizes the most frequent commercial methods, detailing their underlying technology/equipment, regulatory status, molecular markers, assay specifications, turnaround times, and applications.

**Table 3:** Common commercially available ctDNA tests for clinical laboratories.

Test – company	Technology	Equipment	Regulatory status	Molecular markers	Key test specifications^a^	Clinical and research application^a^
Cobas^®^ EGFR Mutation Test v2 – Roche	RT-PCR	Cobas z 480 analyzer	CE-IVD/US-IVD	Mutations/indels * EGFR*	LOD: less than 100 copies of mutant DNA per mL of plasma Turnaround time: 4 h from blood extraction to reporting	Lung cancer
Idylla Mutation Test assays – Biocartis	RT-PCR	Biocartis Idylla™ system	RUO	Mutations *KRAS* *NRAS/BRAF* *EGFR*	LOD < 5 % for all KRAS mutations and for most prevalent EGFR mutations Turnaround time: 3 h from cell-free DNA to results	Lung and colorectal cancer
Therascreen PCR kits – Qiagen	RT-PCR	Rotor-Gene^®^ Q MDx 5plex HRM	CE-IVD/US-IVD/RUO^b^	Mutations *PIK3CA* *EGFR*	Overall percent agreement plasma-tissue 72 % Turnaround time: 1–2 days from blood extraction to reporting	Breast and lung cancer
Plasma-SeqSensei™ Kits – Sysmex	Multiplex PCR – NGS	Illumina NextSeq 500/550 and MiSeq sequencing platforms	CE-IVD/RUO^b^	Mutations/indels Solid cancer kit (*BRAF*, *EGFR*, *KRAS*, *NRAS* and *PIK3CA*) Breast cancer kit (*AKT1*, *ERBB2*, *ESR1*, *KRAS*, *PIK3CA* and T*P53*) NSCLC kit (*EGFR*, *KRAS*, *BRAF* and *PIK3CA*) Colorectal cancer kit (*KRAS*, *NRAS*, *BRAF* and *PIK3CA*)	LOD: 0.06 % MAF Turnaround time: 2 days from cell-free DNA to results, including sequencing time	Solid cancer, lung, colorectal and breast cancer
Oncomine NGS panels – ThermoFisher	NGS – Amplicon-based libraries	Ion GeneStudio S5 system and the ion Torrent Genexus System	RUO	Mutations/fusions/indels/CNVs Pan-cancer cell-free assay (52 genes) Precision assay (50 genes) Lung cfTNA assay (12 genes) Breast cfDNA assay (12 genes) Colon cfDNA assay (14 genes)	LOD: down to 0.1 % VAF for SNV hotspots and indels Turnaround time: 1–3 days from blood extraction to reporting	Pan-cancer, lung, breast, and colorectal cancer
Avenio NGS panels – Roche	NGS – Hybrid-capture based libraries	Illumina NextSeq 500/550 sequencing platform	RUO	Mutations/fusions/indels/CNVs Targeted kit (17 genes) Expanded kit (77 genes) Surveillance kit (197 genes)	LOD: down to 0.5 % VAF Turnaround time: 5 days from cfDNA extraction to reporting	Pan-cancer
TruSight Oncology 500 ctDNA – Illumina	NGS – Hybrid-capture based libraries	Illumina NovaSeq X/NovaSeq 6,000 sequencing platform	RUO	Mutations/fusions/indels/CNVs/MSI/TMB TruSight oncology 500 ctDNA (523 genes)	LOD for small variants: 0.5 % VAF Turnaround time: 3–4 days from purified nucleic acid to variant report	Pan-cancer
Guardant360 CDx – Guardant Health	NGS – Hybridation based capture libraries	Comercial outsourced application	FDA approved	Mutations/indels (74 genes)/fusions (6 genes)/amplifications (18 genes)/MSI	LOD for SNVs varies with cfDNA input: 0.2 % VAF at 30 ng, and 1.8 % VAF at 5 ng Turnaround time: 7 days from sample receipt to results	Solid cancer. Test used to identify patients eligible for targeted therapies.
FoundationONE Liquid CDx – Foundation Medicine	NGS – Hybridation based capture libraries	Comercial outsourced application	FDA approved	Mutations/indels (311 genes)/rearrangements (4 genes)/amplifications (3 genes)/MSI/TMB	Median LOD for short variants ranges from 0.4 to 0.8 % VAF, depending on the genomic region. Turnaround time: 8 days from sample receipt to results	Solid cancer. Test used to identify patients eligible for targeted therapies.
Signatera – Natera	Whole exome sequencing + multiplex PCR-based NGS	Comercial outsourced application	Breakthrough Device Designation	Custom-built assay – based on the unique mutation signature of each patient’s tumor	LOD: 0.01 % VAF Turnaround time: 3 weeks for initial tumor sequencing and personalized assay design; 1–2 weeks for MRD results from sample receipt	Multi-cancer. Test used for treatment monitoring and MRD assessment

^a^According to manufacter specifications. ^b^Regulatory status depends on the specific test. IVD, *in vitro* diagnostic; RUO, research use only; LOD, limit of detection; RT-PCR, real-time PCR; NGS, next-generation sequencing. Gene symbols are shown in italics for emphasis, following common editorial conventions.

Current molecular technologies for ctDNA analysis include PCR-based methods and NGS technologies. PCR-based techniques are designed to identify specific genetic alterations and encompass real-time quantitative PCR (qPCR) and dPCR. The main advantage of these techniques over NGS-based sequencing panels lies in their high sensitivity and specificity for detecting variants, with the ability to identify VAFs at 0.1 % or below [92]. However, PCR-based methods can screen only a limited number of known variants, whereas NGS facilitates simultaneous screening of multiple markers and samples in the same run.

Currently, several commercial qPCR-based products are available. Real-time qPCR tests offer greater ease of use and are designed for specific clinical applications, some of which have been approved for routine clinical use. DPCR-based assays include numerous assays developed for the BioRad QX200/QX600 Droplet Digital PCR System and Thermo Fisher Scientific Absolute Q dPCR System, both of which offer similar sensitivity [93]. However, both reagents and equipment are currently only available for research use only (RUO) and these assays are currently limited to clinical research. Rigorous analytical and clinical validation is indispensable for their use in clinical settings.

NGS-based ctDNA methods allow for the detection of alterations across a broad spectrum of genes. Commercially available NGS panels for ctDNA analysis include the Oncomine NGS assays (Thermo Fisher), Avenio ctDNA kits (Roche), TruSight Oncology 500 ctDNA (Illumina), and QIAseq Targeted cfDNA Ultra Panels (Qiagen), among others. These panels differ in the genes or regions covered, the types of alterations they can detect, and their sensitivity for detecting variants. However, ctDNA mutations above 0.5 % are generally detected by these assays with high sensitivity, precision, and reproducibility [90].

In clinical practice, given the variety of assays currently available for ctDNA analysis, selecting the most appropriate test should be based on availability, reimbursement status, and the number of actionable genetic aberrations within a tumor-specific context [4].

## Comprehensive interpretation of gene variant results in ctDNA assays

Recommendations for identifying, interpreting, and reporting variants in cfDNA analysis should align with established criteria for somatic variant interpretation and oncogenicity classification [94]. However, it is essential to account for the unique characteristics of ctDNA and adhere to the specific guidelines tailored to ctDNA analysis across various tumor types [4], [95].

Variant identification in ctDNA analysis involves detecting SNVs, indels, fusions and CNVs. Although many software tools automate this process, clinical laboratories must be aware of their limitations, as ctDNA analysis presents challenges in accurately for the identification of certain genetic aberrations, such as CNVs or fusion variants [4], [96]. Key metrics, including sequencing depth (coverage) and VAF, are essential for accurate interpretation and should be carefully evaluated [97]. When interpreting ctDNA findings, it is crucial to take into account that these assays have lower sensitivity compared to tissue profiling, which may increase the likelihood of false negative results. It is also important to consider the possibility of false positive results in ctDNA analysis due to the identification of CHIP variants, which can be detected at low VAF (0.1–5%), leading to their misinterpretation as tumor-derived variants [98]. As sporadic benign conditions can contain somatic alterations in cancer driver genes, interpretation of ctDNA assays should be done in the context of clinical information. For example, V600E variant has been found in plasma DNA not only in patients with cancer but also in individuals with benign nevi [99].

It is recommended to classify gene variants by their actionability, using current evidence to guide diagnostics, prognostics, and eligibility for FDA/EMA-approved therapies or clinical trials. In line with this, the Association for Molecular Pathology (AMP) Tier system and the ESMO Scale for Clinical Actionability of molecular Targets (ESCAT) both evaluate genetic alterations based on clinical relevance. AMP tier-based classification categorizes somatic variants into four tiers based on their level of clinical significance: Tier I includes variants with strong clinical relevance, Tier II encompasses variants with potential clinical significance, Tier III includes variants of unknown clinical significance, and Tier IV consists of variants considered benign or likely benign. Levels of evidence A or B (Tier I) and C or D (Tier II) are weighted based on their significance in guiding clinical decision-making [97]. On the other hand, ESCAT categorizes molecular aberrations into Tiers I to V and X, based on the available evidence supporting their value as clinical targets. Tier I include molecular alterations with a recommended specific drug suitable for routine use, while other levels of clinical evidence (ESCAT Tier II to V) require additional data, restricting the clinical application to clinical trials. No clinical or preclinical evidence supports ESCAT Tier X alterations and should not be considered for clinical decisions [100].

Databases like COSMIC, ClinVar, and OncoKB are essential tools for interpreting ctDNA analysis results of patients with cancer (Table 4). These platforms provide context for detected genetic variants by compiling data on somatic variants, pathogenicity, and clinical relevance across different cancer types, contributing also to identify variants of uncertain significance (VUS).

**Table 4:** Common databases used for interpretation of cancer-related gene variants.

Database	Description	URL
Cancer Genome Interpreter (CGI)	Includes tumor alterations that drive the disease and may be therapeutically actionable, relying on computational methods such as in silico saturation mutagenesis of cancer genes (BoostDM and OncodriveMu) [109].	https://www.cancergenomeinterpreter.org
Cancer Hotspots	Provides significant recurrent mutations identified in large-scale cancer genomics data, detected in tumor samples using the described algorithm [110].	https://www.cancerhotspots.org
cBioPortal	Interactive, open-source platform designed for the visualization, exploration, and analysis of genomic cancer data and somatic variants across various tumor types [111].	https://www.cbioportal.org
CiVIC (clinical interpretation of Variants in Cancer)	Provides clinically relevant interpretations of cancer genetic variants to aid therapeutic decision-making, facilitating collaboration among researchers, clinicians, and patients advocates [112].	https://civicdb.org
CKB Core (Jackson Laboratory Clinical Knowledgebase)	Dynamic digital resource for interpreting complex cancer genomic profiles in the context of protein impact, therapies, and clinical trials [113].	https://ckb.jax.org
ClinVar	Public archive cataloging human genetic variations associated with diseases, drug responses, and malignancies; enhancing communication and supporting reevaluation of variant classifications [114]	https://www.ncbi.nlm.nih.gov/clinvar
COSMIC (Catalogue of Somatic Mutations in Cancer)	Source of expert-curated somatic mutation information related to human cancers, offering a comprehensive catalog of somatic variants and associated genes in oncology [115]	https://cancer.sanger.ac.uk/cosmic
DoCM (Database of Curated Mutations)	Curated repository that aggregates gene/variant information for variants with prognostic, diagnostic, predictive, or functional roles from various resources and individual publications [116]	https://docm.info
Franklin	AI-powered platform that automates the workflow from raw sequencing data (FASTQ/VCF) to clinical variant reporting; providing comprehensive variant analysis, literature evidence, automated ACMG-based classification, along with annotations and assessment tools [117].	https://franklin.genoox.com
My Cancer Genome	Provides insights into the clinical impact of molecular biomarkers on drug use in oncology, based on FDA labels, NCCN guidelines, clinical trials, and peer-reviewed publications, using data from tumor samples in the AACR project GENIE database [118].	https://www.mycancergenome.org
PMKB (Precision Medicine Knowledgebase)	An interface for collaborative editing and sharing of clinical-grade cancer mutation interpretations, designed to support the collection, maintenance, and reporting of interpretations for clinical cancer genomic testing [119].	https://pmkb.weill.cornell.edu
OncoKB	Focuses on precision oncology, providing biological and clinical data on genomic alterations in cancer. Alterations and tumor type-specific therapeutic implications are classified using the OncoKB™ levels of evidence system [120]	https://www.oncokb.org
VarSome Clinical	A platform for variant discovery, annotation, and interpretation of NGS data, integrating public databases and algorithms to provide detailed information on variant pathogenicity, population frequency, and clinical significance [121].	https://clinical.varsome.com/

AACR, American Association for Cancer Research; ACMG, American College of Medical Genetics and Genomics; FDA, Food and Drug Administration; NCCN, National Comprehensive Cancer Network; NGS, Next-Generation Sequencing.

To accurately interpret gene variants detected in ctDNA assays, it is essential to establish tumor molecular boards composed of a diverse team of healthcare professionals. Laboratory professionals within these multidisciplinary teams are crucial for evaluating the clinical relevance of molecular findings. They ensure that genetic alterations are interpreted accurately and in the context of the patient’s overall clinical scenario. Molecular tumor boards offer critical insights, particularly in complex cases with uncertain or conflicting data. These collaborative efforts enhance the quality of patient care by integrating various perspectives and expertise, ultimately leading to better treatment outcomes [101].

## Clinical applications of ctDNA

### Currently recommended applications: advanced disease

In clinical practice, ctDNA assays are considered reliable for genotyping advanced cancers and directing molecularly targeted therapies, especially in situations where tissue biopsies are suboptimal, or time is crucial [4]. The clinical utility of these assays in guiding therapy for Tier I actionable variants is supported by recent large prospective ctDNA-based studies, which have demonstrated high accuracy for SNVs (referring to tissue-plasma comparisons) across various types of cancer [27], [102], [103], [104].

In ctDNA assays, high sensitivity is achieved for SNVs and small indels. However, other aberrations such as fusions, CNVs, or microsatellite instability (MSI) may exhibit reduced sensitivity and should only replace tissue assessment when tissue testing is not feasible [4], [104]. In this context, a negative result for an actionable genetic alteration should be considered non-informative if there is no additional evidence of sufficient ctDNA levels in the assay. In such cases, confirmation with tissue testing is recommended [4], [105]. While tumor mutation burden (TMB) has shown potential as a predictive biomarker for immunotherapy, it remains an area of ongoing research [4], [106].

Nowadays, general recommendations for the use of ctDNA across various tumor types primarily target patients who lack tissue-based genomic test results when genomic testing is indicated, archival tissue is unavailable, or new tumor biopsies are not feasible [4]. Table 5 presents specific ESMO recommendations for the use of ctDNA assays in routine clinical practice, including Tier I actionable molecular markers (ESCAT scale) and associated FDA-approved drugs.

**Table 5:** CtDNA applications of Tier I variants (ESCAT) in clinical setting for advanced cancer disease.

Tumor type	Gene	Aberrations	Drugs/therapy^a^		ESMO recommendation for ctDNA analysis [4]
Non-small cell lung cancer	*EGFR*	T790M mutation	Osimertinib		Genotyping recommended in treatment-naïve cancer patients and resistance upon prior TKIs. Fusion detection is suboptimal and should be repeated in tissue where possible
	*EGFR*	Exon 19 in-frame deletions, L858R	Erlotinib, Erlotinib + Ramucirumab, Afatinib, Dacomitinib, Gefitinib, Osimertinib, Amivantamab + Lazertinib		
	*EGFR*	Exon 20 in-frame insertions (762_823ins)	Amivantamab		
	*EGFR*	G719, S768I, L861Q mutations	Afatinib		
	*ALK*	Fusions	Alectinib, Brigatinib, Ceritinib, Crizotinib, Lorlatinib		
	*MET*	D1010, exon 14 deletion, exon 14 in-frame deletions, exon 14 splice mutations	Capmatinib, Tepotinib		
	*KRAS*	G12C	Sotorasib, Adagrasib		
	*BRAF*	V600E	Dabrafenib + Trametinib, Encorafenib + Binimetinib		
	*RET*	Fusions	Selpercatinib, Pralsetinib		
	*ROS1*	Fusions	Crizotinib, Entrectinib, Repotrectinib		
	*NTRK 1/2/3*	Fusions	Entrectinib, Larotrectinib, Repotrectinib		
	*NTRK 1/2/3*	Acquired resistance mutations	Entrectinib, Larotrectinib		
Colorectal cancer	*BRAF*	V600E	Encorafenib + Cetuximab		*KRAS/NRAS/BRAF* ^V600E^/MSI for chemotherapy-naive metastatic colorectal cancer when tissue not available or urgent therapeutic decision making. *KRAS/NRAS/BRAF/EGFR*-ECD for pretreated patients if *EGFR* rechallenge is planned
	*MSI-H*	Microsatellite instability-high (MSI-H)	Pembrolizumab, Nivolumab, Ipilimumab + Nivolumab		
	*NTRK 1/2/3*	Fusions	Entrectinib, Larotrectinib, Repotrectinib		
	*KRAS/NRAS*	Exon 2,3,4 mutations	Cetuximab, Panitumumab		
	*KRAS*	G12C	Adagrasib + Cetuximab		
	*ERBB2*	Amplification	Tucatinib + Trastuzumab		
	*EGFR*	Mutations in the extracellular domain S492, G465, S464, V441	Cetuximab, Panitumumab		
Pancreatic and hepatocellular cancer	MSI-H	Microsatellite instability-high (MSI-H)	Pembrolizumab		When tissue not available
	*NTRK 1/2/3*	Fusions	Entrectinib, Larotrectinib, Repotrectinib		
Gastric cancer	*ERBB2*	Amplification	Pembrolizumab + trastuzumab + chemotherapy, trastuzumab + chemotherapy, trastuzumab deruxtecan		When tissue not available or urgent therapeutic decision making
	MSI-H	Microsatellite instability-high (MSI-H)	Pembrolizumab		
	*NTRK 1/2/3*	Fusions	Entrectinib, Larotrectinib, Repotrectinib		
Breast cancer	*PIK3CA*	C420R, E542K, E545A, E545D, E545G, E545K, H1047L, H1047R, H1047Y, Q546E, Q546R mutations	Pembrolizumab Trastuzumab + Chemotherapy, Trastuzumab + Chemotherapy, Trastuzumab Deruxtecan		*ESR1* mutations should preferentially be tested in ctDNA. *ERBB2* amplification and NTRK fusions when tissue not available
	*ERBB2*	Amplification	Ado-trastuzumab emtansine, Lapatinib + Capecitabine, Lapatinib + Letrozole, Margetuximab + Chemotherapy, Neratinib, Neratinib + Capecitabine, Trastuzumab, Trastuzumab + Chemotherapy, Trastuzumab + Pertuzumab + Chemotherapy, Trastuzumab + Tucatinib + Capecitabine, Trastuzumab Deruxtecan		
	*ESR1*	D538 and E380, L469V, L536, S463P, Y537	Elacestrant		
	MSI-H	Microsatellite instability-high (MSI-H)	Pembrolizumab		
	*NTRK 1/2/3*	Fusions	Entrectinib, Larotrectinib, Repotrectinib		
Colangiocarcinoma	*IDH1*	R132 mutations	Ivosidenib		When tissue not available or urgent therapeutic decision making.
	*FGFR2*	Fusions	Futibatinib, Pemigatinib		
	MSI-H	Microsatellite instability-high (MSI-H)	Pembrolizumab		
	*NTRK 1/2/3*	Fusions	Entrectinib, Larotrectinib, Repotrectinib		
Ovarian cancer	*BRCA1/2*	Mutations	Olaparib, Olaparib + Bevacizumab, Niraparib, Rucaparib		In women with no germline pathogenic *BRCA1/2* variant when tissue not available
	MSI-H	Microsatellite instability-high (MSI-H)	Pembrolizumab		
Endometrial cancer	MSI-H	Microsatellite instability-high (MSI-H)	Pembrolizumab		When tissue not available
Prostate cancer	*BRCA1/2*	Mutations	Olaparib, Olaparib + Bevacizumab, Niraparib, Rucaparib		When tissue not available
	MSI-H	Microsatellite instability-high (MSI-H)	Pembrolizumab		
Urothelial cancer	*FGFR*	G370C, R248C, S249C, Y373C mutations	Erdafitinib		When tissue not available.
	*FGFR3*	Fusions	Erdafitinib		
	*NTRK 1/2/3*	Fusions	Entrectinib, Larotrectinib, Repotrectinib		
Thyroid cancer	*BRAF*	V600E	Dabrafenib + Trametinib		When tissue not available.
	*RET*	Mutations and fusions	Pralsetinib, Selpercatinib		
	*NTRK 1/2/3*	Fusions	Entrectinib, Larotrectinib, Repotrectinib		
Soft tissue sarcoma	*NTRK 1/2/3*	Fusions	Entrectinib, Larotrectinib, Repotrectinib		When tissue not available

^a^FDA-approved related drugs defined in OncoKB [120] in October 2024. Gene symbols are shown in italics for emphasis, following common editorial conventions.

Beyond the currently recommended use of ctDNA in routine clinical practice, recent studies further support other clinical utilities in the context of advanced disease. In this regard, ctDNA tumor fraction has been established as an independent prognostic biomarker across multiple cancers [107], and ctDNA molecular profiling has shown utility in selecting patients for early-phase targeted therapies [108].

### Potential applications

Despite recent advances, there are clinical applications of liquid biopsy that are not yet routinely implemented in the clinic. This is the case for example of early diagnosis/screening, detection of minimal residual disease (MRD), and monitoring of disease during treatment. Although ctDNA assays can improve diagnostic processes and help identify early-stage cancers, several challenges need to be resolved for their implementation in the clinic [4]. Achieving high specificity and clinically relevant sensitivity is difficult, particularly because early-stage cancers release low levels of ctDNA [55]. To effectively implement ctDNA assays in clinical practice as validated screening tools, large population studies are needed [4]. In line with this, recent studies are increasing the evidence for using ctDNA for early detection/screening of patients with cancer [5], [122], [123].

Regarding the detection of MRD, the analysis of ctDNA after curative treatment in early-stage cancers predicts a high risk of relapse with high clinical specificity [124]. In recent years, interest in MRD has grown significantly, leading to ctDNA-guided randomized clinical trials in colorectal, lung, and breast cancer, which are yielding promising results for the implementation of ctDNA in MRD assessment. In this context, post-surgical ctDNA monitoring in resectable colorectal cancer has proven useful for identifying patients at high risk of recurrence and/or mortality, who are more likely to benefit from adjuvant chemotherapy [66], [125]. Furthermore, serial ctDNA analysis in patients with colon cancer undergoing adjuvant therapy enables treatment escalation or de-escalation, allowing for a more precise selection of patients who truly benefit from adjuvant therapy compared to the conventional tumor/node/metastasis (TNM) staging system [126]. Notably, a recent study in localized colon cancer demonstrated that MRD prediction accuracy can be enhanced by using NGS panels that track multiple ctDNA gene variants across serial plasma samples [127]. In early-stage non-small cell lung cancer, the detection of residual ctDNA after treatment has shown utility in predicting early relapse [128], and in breast cancer, ctDNA profiling is able to detect the onset of recurrences [129].

The use of ctDNA has also shown promise for monitoring treatment responses and resistance development in patients with cancer [21]. Its short half-life and possibility of real-time sampling, make ctDNA valuable for assessing disease dynamics during therapy [4]. Studies indicate that ctDNA levels correlate with treatment response and can detect changes earlier than traditional clinical methods [21], [130]. However, ctDNA is not yet implemented in clinical settings due to several limitations, such as the need for optimal assay strategies, uncertainties about monitoring frequency, and insufficient evidence of improvements in patient outcomes [4].

## Reporting of gene variants detected in ctDNA

Generating reports from molecular testing is essential for translating complex genetic data into useful clinical information. These reports should have a standardised format, clearly state the date of issue, and include diagnosis details and significant medical information when available. They should be clear and concise, presenting clinically significant information in an easily understandable way. Additionally, reports should be formatted for easy integration with electronic health records. Clinically critical information must be placed at the beginning for quick access, and more complex data should be simplified using graphs, charts, and tables [97].

Genetic alterations should be thoroughly described, including the involved genes, the type of variants or genomic features detected (such as SNVs, indels, CNVs, and fusions), and their predicted impact on protein function. Adopting standardized nomenclature according to Human Genome Variation Society (HGVS) guidelines (http://varnomen.hgvs.org/) is essential to avoid confusion and clinical errors [94]. The report should include relevant elements for thorough analysis and longitudinal comparisons, such as genomic coordinates, the genome build, and the transcript reference sequence. Including the VAF in reports, whenever possible with quantitative assays, provides critical insights for evaluating the reliability of detected variants, particularly regarding the risk of false negatives.

Following the AMP-Tier system, it is recommended to report variants classified as Tier I to III in order of their clinical significance. Generally, Tier IV variants, categorized as benign or likely benign, should not be included. Interpretative comments should be provided, particularly for Tiers I and II gene variants. Recommendations should be evidence-based and supported by appropriate literature citations [97]. Clinical actionability annotation is a crucial component of the report, supporting the clinical interpretation of results. Only likely pathogenic or pathogenic oncogenic driver alterations should be assessed for clinical actionability using clinical evidence-based frameworks such as ESCAT, OncoKB classification system, or AMP tier classification [131].

On the other hand, when a gene variant is not detected, it is preferable to use terms such as ‘non-informative’ or ‘not detected’ instead of ‘negative’ [4]. The report should acknowledge the potential for discrepancies with tumor testing, especially when no variant is found in plasma DNA.

The analysis of ctDNA could identify incidental germline variants. In the case of reporting these incidental findings, it would be convenient to clearly differentiate between somatic and putative germline variants, as well as include information about the need to perform confirmatory tests in peripheral blood leukocytes, or in other normal tissue samples [78], [79].

Methodological details and limitations should be included at the end of the report, covering the alterations tested, assay performance characteristics [such as the limit of detection for each variant type and minimal sequencing depth), and critical quality metrics [86]. Information on any preanalytic, analytic, or postanalytic factors that might influence clinical interpretation should be indicated. It is important to note that assay sensitivity may depend on the amount of input cfDNA. Therefore, when plasma cfDNA is limited, the reported sensitivity may be adjusted or a warning included in the report [86].

## Future perspectives of ctDNA

In addition to the study of gene variants, one of the most promising areas of ctDNA research are epigenomics (DNA methylation) and fragmentomics. These fields have demonstrated great promise for the early detection of cancer, the identification of tumor origin, and the evaluation of therapy response [5], [35]. It is also important to highlight that artificial intelligence, particularly through machine learning algorithms, is starting to play a crucial role in the discovery and implementation of new ctDNA biomarkers. In addition to enabling more precise and comprehensive analyses of genomics, epigenomics, and fragmentomics [132], artificial intelligence also facilitates the integration of these omics approaches with clinical data, driving further advancements in personalized cancer care [133].

Despite these advances, several challenges need to be addressed before ctDNA can be widely integrated into clinical practice. One major issue is sensitivity, particularly in early-stage cancer where ctDNA levels are typically low. Improved sensitivity in ctDNA assays, achieved through methods such as NGS and dPCR, represents a key area for future research [3], [134].

The potential for ctDNA to provide real-time insights into tumor heterogeneity is a relevant advantage. By capturing spatial and temporal genomic variations within a patient, ctDNA can offer a more comprehensive picture of tumor evolution than traditional tissue biopsies. This can be particularly beneficial in advanced cancer stages where tumors often exhibit significant heterogeneity, contributing to treatment resistance [4], [124]. However, false positives remain a concern, especially when ctDNA mutations overlap with CHIP [75], underscoring the need for robust assay development and validation.

The clinical utility of ctDNA is also gaining traction in the context of MRD detection. Monitoring ctDNA levels post-treatment could help identify patients at risk of relapse, allowing for timely therapeutic intervention [4]. Ongoing clinical trials are expected to provide critical data on the role of ctDNA in MRD, tracking tumor evolution, and guiding treatment decisions [66].

To further enhance the diagnostic utility of ctDNA, it is crucial to standardize both preanalytical and analytical procedures. Initiatives such as the BloodPac in the United States and Cancer-ID in Europe are actively working on establishing standard operating procedures for ctDNA analysis [135], [136]. Standardization will not only improve reproducibility but also facilitate the large-scale clinical implementation of ctDNA testing.

Another promising area of research lies in combining ctDNA analysis with other circulating biomarkers, such as circulating tumor cells (CTCs) and extracellular vesicles (EVs). This approach could provide more comprehensive insights into tumor biology, helping to refine diagnosis and guide treatment decisions [1]. On the other hand, the study of ctDNA in biological fluids beyond plasma is gaining significant attention for its potential to improve cancer management in certain types of tumors [46], [52], [137].

Despite these advancements, the translation of ctDNA into clinical practice remains limited due to several technical and economic barriers. Current NGS-based approaches, although highly sensitive, require sophisticated laboratory equipment and are time-consuming, making them difficult to implement on a large scale [138]. Further technological developments, including the creation of more cost-effective and user-friendly assays, will be essential to overcome these limitations [3].

## Conclusions

The analysis of ctDNA is paving the way for a more personalized cancer care. Over the past decade, advancements in ctDNA technology have been substantial, with numerous studies highlighting its potential to revolutionize the management of patients with cancer [1]. For patients with advanced cancer, validated and adequately sensitive ctDNA assays have nowadays utility in identifying actionable mutations to direct targeted therapy, and may be used in routine clinical practice, particularly when rapid results are needed or when tissue biopsies are not possible or inappropriate. In addition, ctDNA analysis offers significant potential for cancer diagnostics, detection of MRD, monitoring, and evaluation of therapy response [4].

In summary, ctDNA analysis offers significant potential for advancing early cancer detection and personalized treatment approaches, which will significantly improve patient outcomes. However, widespread clinical implementation will require further validation, standardization, and cost-reduction strategies. As ongoing trials continue to yield valuable insights, it is likely that ctDNA, combined with other circulating biomarkers, will become a cornerstone of modern clinical laboratories.
